# Supplementary material for: The causal role of circulating inflammatory markers in osteoporosis: a bidirectional Mendelian randomized study
Source: Front Immunol. 2024 Jul 18;15:1412298. doi: 10.3389/fimmu.2024.1412298 (PMC11291241; doi:10.3389/fimmu.2024.1412298)
Supplement: Supplementary file 1 [file DataSheet_1.zip › supplementary material/OSTEOPOROSIS-CIM/GCST90274784.tsv.gz_forest.pdf]

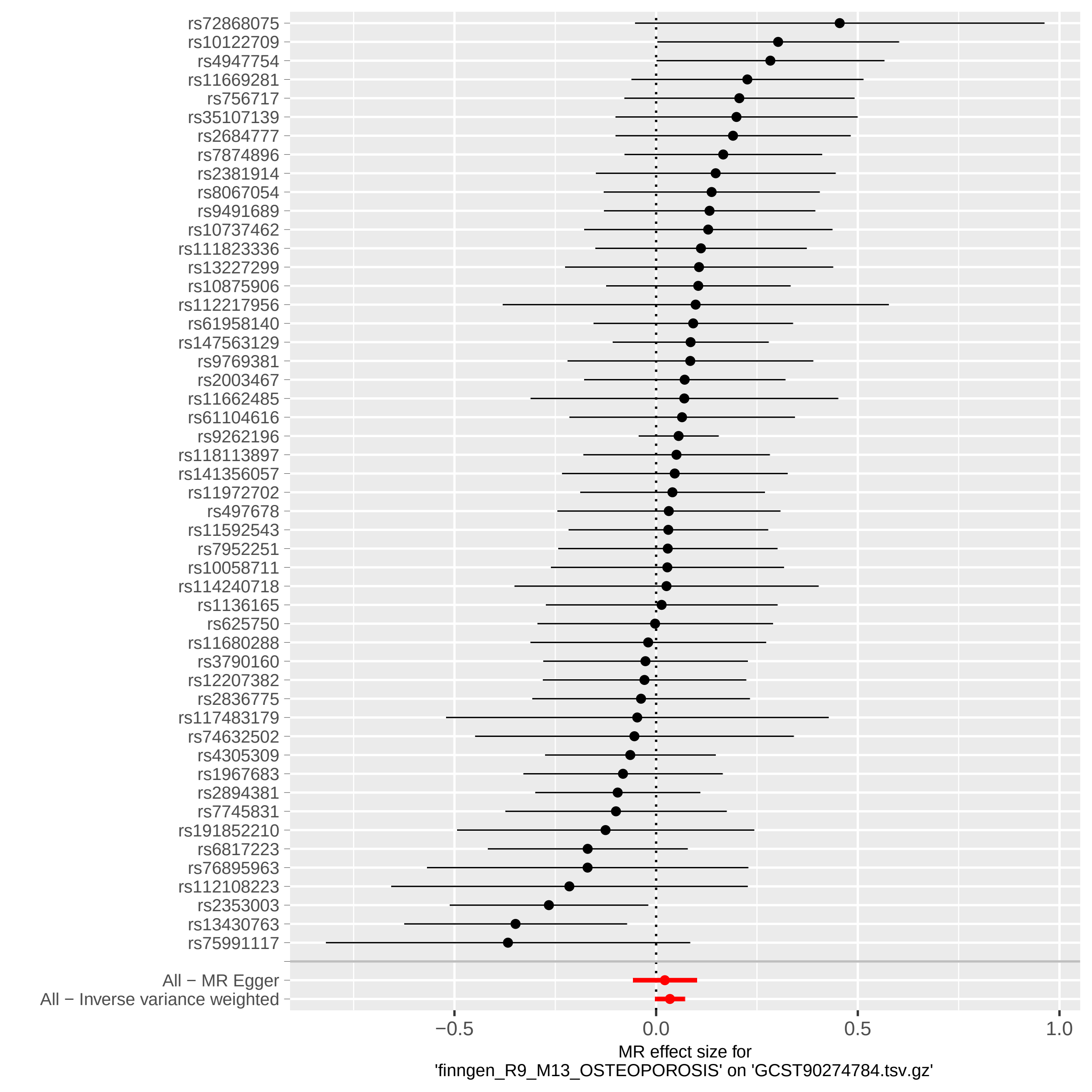
rs72868075
rs10122709
rs4947754
rs11669281
rs756717
rs35107139
rs2684777
rs7874896
rs2381914
rs8067054
rs9491689
rs10737462
rs111823336
rs13227299
rs10875906
rs112217956
rs61958140
rs147563129
rs9769381
rs2003467
rs11662485
rs61104616
rs9262196
rs118113897
rs141356057
rs11972702
rs497678
rs11592543
rs7952251
rs10058711
rs114240718
rs1136165
rs625750
rs11680288
rs3790160
rs12207382
rs2836775
rs117483179
rs74632502
rs4305309
rs1967683
rs2894381
rs7745831
rs191852210
rs6817223
rs76895963
rs112108223
rs2353003
rs13430763
rs75991117
All − MR Egger
All − Inverse variance weighted
−0.5
0.0
0.5
1.0
MR effect size for
'finngen_R9_M13_OSTEOPOROSIS' on 'GCST90274784.tsv.gz'
